# Racial Discrimination, Sedentary Time, and Physical Activity in African Americans: Quantitative Study Combining Ecological Momentary Assessment and Accelerometers

**DOI:** 10.2196/25687

**Published:** 2021-06-07

**Authors:** Soohyun Nam, Sangchoon Jeon, Garrett Ash, Robin Whittemore, David Vlahov

**Affiliations:** 1 School of Nursing Yale University West Haven, CT United States; 2 Veterans Affairs Connecticut Healthcare System West Haven, CT United States; 3 Center for Medical Informatics Yale University New Haven, CT United States

**Keywords:** racial discrimination, physical activity, ecological momentary assessment, African American, pilot study, mobile phone

## Abstract

**Background:**

A growing number of studies indicate that exposure to social stress, such as perceived racial discrimination, may contribute to poor health, health behaviors, and health disparities. Increased physical activity (PA) may buffer the impact of social stress resulting from racial discrimination. However, to date, data on the relationship between racial discrimination and PA have been mixed. Part of the reason is that the effect of perceived racial discrimination on PA has primarily been examined in cross-sectional studies that captured retrospective measures of perceived racial discrimination associated with individuals’ current PA outcomes. The association between real-time perceived racial discrimination and PA among African Americans remains unclear.

**Objective:**

The purpose of this study is to examine the relationship among demographic, anthropometric and clinical, and psychological factors with lifetime racial discrimination and examine the within- and between-person associations between daily real-time racial discrimination and PA outcomes (total energy expenditure, sedentary time, and moderate-to-vigorous PA patterns) measured by ecological momentary assessment (EMA) and accelerometers in healthy African Americans.

**Methods:**

This pilot study used an intensive, observational, case-crossover design of African Americans (n=12) recruited from the community. After participants completed baseline surveys, they were asked to wear an accelerometer for 7 days to measure their PA levels. EMA was sent to participants 5 times per day for 7 days to assess daily real-time racial discrimination. Multilevel models were used to examine the within- and between-person associations of daily racial discrimination on PA.

**Results:**

More EMA-reported daily racial discrimination was associated with younger age (*r*=0.75; *P*=.02). Daily EMA-reported microaggression was associated with depressive symptoms (*r*=0.66; *P*=.05), past race-related events (*r*=0.82; *P*=.004), and lifetime discrimination (*r*=0.78; *P*=.01). In the within-person analyses, the day-level association of racial discrimination and sedentary time was significant (β=.30, SE 0.14; *P*=.03), indicating that on occasions when participants reported more racial discrimination than usual, more sedentary time was observed. Between-person associations of racial discrimination (β=−.30, SE 0.28; *P*=.29) or microaggression (β=−.34, SE 0.36; *P*=.34) with total energy expenditure were suggestive but inconclusive.

**Conclusions:**

Concurrent use of EMA and accelerometers is a feasible method to examine the relationship between racial discrimination and PA in real time. Examining daily processes at the within-person level has the potential to elucidate the mechanisms of which racial discrimination may have on health and health behaviors and to guide the development of personalized interventions for increasing PA in racial ethnic minorities. Future studies with a precision health approach, incorporating within- and between-person associations, are warranted to further elucidate the effects of racial discrimination and PA.

**International Registered Report Identifier (IRRID):**

RR2-10.1002/nur.22068

## Introduction

### Background

African Americans continue to experience disproportionately higher rates of cardiovascular disease and metabolic disorders than their White counterparts [1]. Although health disparities have been attributed to multiple factors, African Americans have been more likely than other racial and ethnic groups to report perceived racial discrimination (eg, 71.3% vs 24% in non-Hispanic Whites) [2-4]. In extensive research, exposure to racial discrimination events or perceived racial discrimination contributes to poor health, health behaviors, and health disparities [4,5].

Social stress derived from systems of inequality, such as racial discrimination, may provoke severe psychological and physiological responses and has been associated with unhealthy behaviors [6,7]. Studies have shown that perceived racial discrimination is linked to the consumption of fatty foods, smoking, and alcohol intake [4,8].

Increased physical activity (PA) may buffer the impact of social stress resulting from racial discrimination [9,10]. To date, studies on the relationship between racial discrimination and PA have shown inconclusive findings. For example, in a multiethnic study of PA, racial discrimination was not associated with PA as measured by pedometers when examined among the full sample or separately by race and ethnicity [11]. An unexpected finding was reported in the Jackson Heart Study cohort [12], with higher daily and lifetime racial discrimination associated with more PA in women based on their self-reported PA.

In addition, although not in the context of racial discrimination, some studies of psychological stress in other populations have linked perceived stress with less PA [13,14]. In recent studies that examined both between- and within-person effects of daily stress on PA, there was significant between-person variability in the relationship between PA and stress [15,16]. For example, the relationship may be bidirectional for some people; for others, it may be unidirectional or have no association, suggesting that examining the within-person effect of stress on PA may address the limitations of between-person analysis that predominate in traditional research [15,16].

To date, data on the relationship between racial discrimination and PA are sparse and inconsistent. Part of the reason is that the literature to date on the effect of perceived racial discrimination on PA comprises mostly cross-sectional studies that capture retrospective measures of lifetime discrimination associated with individuals’ current health outcomes. Such data may be subject to recall and rumination biases. Furthermore, racial microaggressions—the brief and commonplace daily verbal or nonverbal denigrating messages directed toward racial and ethnic minorities that carry the offending party’s implicit or unconscious bias—have been shown to disempower racial minorities and may negatively impact health outcomes [17,18]. However, this subtle form of racial discrimination may be difficult to capture by retrospective measures and has been understudied in research on perceived racial discrimination and health. In this study, we prospectively examined racial microaggression (hereinafter microaggression) as a subtle form of racial discrimination as well as lifetime racial discrimination. Examining perceived racial discrimination or microaggression at a single point in time—not incorporating the perspective that this experience fluctuates but combining with cumulative past experience of racial discrimination—may have limitations in examining the differences in behavioral responses across settings and time.

Ecological momentary assessment (EMA) is a real-time, self-report data-capturing method in which people report behavior in real time at multiple time points in their natural environment. It may reduce recall biases and enhance ecological validity by collecting self-report data that are more proximal to the time and place (ie, real world) in which stressful events and behaviors occur [19]. Recently, a growing number of studies that explore discrimination and health outcomes using EMA have been published; for example, the relationships between real-time discriminatory experiences and health behaviors have been examined in various sexual and gender ethnic minority groups [20-22]. The EMA method provides the opportunity to examine how fluctuations in daily perceived racial discrimination or microaggressions are associated with PA among African Americans at the within-person level. In addition, the use of accelerometers can minimize the weakness of self-report measures of PA.

### Objectives

Therefore, the purpose of this pilot study is (1) to describe the relationship among demographic (age, sex, income, and education), anthropometric and clinical factors (BMI, blood pressure, and body composition), and psychological factors (depression) with lifetime racial discrimination and (2) to examine the effects of real-time racial discrimination on total energy expenditure, sedentary time, and moderate-to-vigorous physical activity (MVPA) patterns of objectively measured PA using accelerometers and a real-time data capture strategy, that is, EMA in healthy African American adults at both the group (eg, between-person level) and individual (eg, within-person level approach or N-of-1) level.

## Methods

### Study Design

This study is a substudy of an intensive, observational, case-crossover design to examine the effects of perceived racial discrimination on physiological (ie, stress biomarkers) and behavioral responses in African Americans. Details of the overall study protocols have been published elsewhere [23]. In a case-crossover design, each participant serves as their own control to assess the within-person effects on repeatedly measured PA outcomes [24]. Within-person analysis of effects on PA occurred at the 2-hour interval level (using EMA at the end of the interval querying participants about racial discrimination over the duration of the interval) and day level (using average scores of EMA response across the day).

### Participants and Recruitment

Building on a relationship developed over the past 10 years, the research team recruited participants from greater New Haven communities in Connecticut via flyers and word-of-mouth communication within African American communities. Before implementing the study, we held meetings with community stakeholders to discuss an effective recruitment plan and the details of the pilot study protocols. Potential participants were called in and were screened by phone and scheduled for a baseline orientation visit. The inclusion criteria were (1) self-reported African American or Black, (2) aged between 30 and 55 years, (3) currently employed, (4) ownership of a smartphone, (5) able to respond to smartphone-based random survey prompts (ie, EMA) at least 3 times per day, and (6) English speaking. We excluded participants who were pregnant or who had serious acute or terminal medical conditions that would preclude PA. The sample size (n=12) was largely based on guidelines for pilot studies that suggest 10 to 40 participants per cell [25]. Even assuming moderate attrition of 20% (2/12), we would have 10 subjects, which is still within the guidelines for pilot studies [26]. We also estimated the minimum detectable effect sizes of other outcomes (ie, stress biomarkers—data not shown; [23]). Our observations would be able to detect medium effect sizes of 0.53-0.60 on primary outcomes (stress biomarkers) repeatedly measured within the individual with 80% power at a 5% significance level, based on a previous study using stress biomarkers [27].

### Baseline Measures

Baseline surveys included sociodemographic characteristics; current smoking status (yes or no); and alcohol consumption by the Alcohol Use Disorders Identification Test [28], which includes frequency of drinking and amount of alcohol consumption. We also used validated self-report measures collected at baseline that are mentioned below.

Perceived racial discrimination was measured at baseline using 2 scales. The *Major Life Discrimination* (MLD) scale is a 9-item self-report measure of past exposures to lifetime discrimination in diverse domains. Respondents indicated whether they had ever experienced each listed major discrimination event (eg, denied a bank loan, unfairly fired, getting a job, at work, and stopped by police; Cronbach α≥.88) [29]. The MLD score represented the sum of each yes or no item (range 0-9). Higher scores indicate more lifetime discriminatory experiences. The *Race-Related Events Scale* (RES) has 22 items to assess exposure to stressful and potentially traumatizing experiences of race-related stresses in adults. Respondents indicated whether they had ever experienced each event (yes or no), and the items were summed for a total RES score ranging from 0 to 22 (Cronbach α=.78-.88) [30]. Higher scores indicate more experiences of race-related stressful events.

The Black Racial Identity–Centrality subscale (Cronbach α>.77) is an 8-item, 7-point Likert scale (ranging from strongly disagree=1 to strongly agree=7). The centrality dimension of racial identity refers to the extent to which individuals normally define themselves with regard to race. It is a measure of whether race is a core part of an individual’s self-concept [31]. After reverse-scoring 3 items, the overall score was calculated by averaging all items, with higher scores indicating stronger racial identity.

For *subjective social status*, participants were asked to place an “X” on the rung that best represented where they thought they stood on the ladder, with 10 rungs described as follows: at the top of the ladder are people who are the best off, those who have the most money, those who have the most education, and those who have the best jobs, and at the bottom are people who are the worst off, those who have the least money, those who have the least education, and those who have the worst jobs or no job (test-retest reliability, *ρ*=0.62) [32,33].

The Center for Epidemiological Studies Depression Scale (CES-D) is a 4-point Likert scale that captures current depressive symptoms with 20 items on how respondents have felt or behaved during the past week by selecting 1 of the 4 options (0=rarely, 1=some of the time, 2=occasionally, and 3=most of the time). The items were summed to obtain a total score. Higher numbers indicate greater depressive symptoms (Cronbach α>.85) [34]. A recent meta-analysis [35] showed that a cutoff point of 20 yields a more adequate trade-off between sensitivity and specificity, compared with the cutoff point of 16, which has been used to indicate probable clinical depression.

### EMA Measures

Perceived racial discrimination was measured using the Experiences of Discrimination (EOD; Cronbach α>.88) [29] and Racial Microaggression Scale (RMAS; Cronbach α>.85) [36,37] adapted for EMA data collection. The EOD has subscales for worry, global, filed complaint, response to unfair treatment, day-to-day discrimination, and skin color [29]. The RMAS has subscales for invisibility, criminality, low-achieving or undesirable culture, sexualization, foreigner or not belonging, and environmental invalidations [36]. As the EOD and RMAS measure experiences of unfair treatment over the past month to year, of which response options are not relevant for real-time EMA assessment, response choices were revised for the EMA time frame using yes or no answers or Likert scale options. We also used a random subscale inclusion strategy so that only 60% of the items would be included in each EMA survey to reduce the subject burden and survey fatigue [38]. When prompted, participants were asked to report whether they had experienced any unfair treatment from a list of 11 common daily racial discriminations since their last prompt or within the past 2-3 hours if they missed or did not complete their last prompt (eg, “treated with less courtesy than other people because of your race or ethnicity,” yes=1 or no=0) and also from a list of 32 microaggression experiences (eg, “people mistake me for being a service worker simply because of my race or ethnicity,” 1=strongly disagree to 7=strongly agree). Possible daily scores of the EOD range from 0 to 10, with higher scores indicating more racial discriminatory experiences. Possible daily RMAS scores range from 15 to 105, with higher scores indicating more microaggression. Each survey (5 times per day) consisted of 8-15 different combinations of questions varying by time of day (sequentially from the first survey to the fifth survey throughout the day).

### PA Measures

PA was measured using a triaxial hip accelerometer (ActiGraph GT9X), which samples movement at 30 Hz and aggregates data into 60-second epochs. The intensity cut points for PA were defined using validated thresholds for vertical axis accelerometry (sedentary<100 counts/min, moderate=2020 counts/min, and vigorous=5999 counts/min) [39]. Energy expenditure was calculated using respective validated triaxial vector magnitude (VM) equations for >2453 VM counts per minute [40] and ≤2453 VM counts per minute [41]. The nonwear periods were defined as ≥60 consecutive minutes of zero activity intensity counts, with allowance for 1-2 minutes of counts between 0 and 100. We considered a day valid if ≥10 hours of activity counts were collected [39] and a 2-hour interval valid if the full time was collected. Accelerometer data were downloaded into ActiLife software (ActiGraph) using the software’s normal filters and scored to create the following variables: total wear time (min), daily wear time (hour/day), total daily energy expenditure, MVPA (min/day), and sedentary time (hour/day). For within-person analyses, these were normalized to the wear time (eg, percent time in MVPA).

### Procedures

Institutional review board approval was obtained from Yale University, and written informed consent was obtained from all participants. At the initial study visit, face-to-face baseline interviews were completed using validated questionnaires. Body weight and height were measured using a portable electronic scale (Omron HBF-514C body composition monitor and scale) and a stadiometer (Seca) following standard procedures. BMI was calculated as weight (kg)/height squared (m^2^). Percent body composition was measured using the same digital scale that measures foot-to-foot bioelectric impedance. This method has demonstrated significant correlations with the gold standard of body fat calculation (ie, dual energy x-ray absorptiometry scan) [42]. After 5 minutes of rest, blood pressure was measured twice with an automated cuff (Omron HEM 780 IntelliSense automatic blood pressure monitor), with 1 minute between readings, and the average of the 2 readings was recorded.

To tailor the EMA survey delivery time, we asked participants for their sleep, wake, or commuting schedules by phone before the baseline study visit. At the baseline visit, we loaded the mEMA app, which is compatible with both iOS and Android operating systems, into each participant’s smartphone. The EMA survey prompted each participant at a random time within the 5 preprogrammed windows daily (ie, signal-contingent sampling) for 7 days (a total of 35 signals) to ensure adequate spacing throughout the day, except for nighttime and commuting time. Upon hearing the signal or vibration, the participants were instructed to complete a short electronic question sequence using their smartphone. Each EMA survey took approximately 3-4 minutes to answer. The EMA data collection system recorded the date and time it took each participant to respond to a random prompt survey and the date and time the survey expired. The survey expired after 40 minutes of nonresponse. After no entry was made, the EMA program became inaccessible until the next recording opportunity.

Participants were instructed to wear an accelerometer on their right hip during waking hours for 7 consecutive days to obtain at least three weekdays and one weekend day to determine the daily variability [39,43]. A paper diary was provided, and participants were instructed to fill out the diary on the time they took off (eg, shower) and wore their accelerometers. All participants received one-on-one in-person training in the EMA surveys and accelerometers. We also provided pictures and step-by-step written instructions on the use of EMA, accelerometers, a tiered payment schedule, and research staff contact information. In addition to the study questions, we sent reminders through EMA to wear their accelerometer daily for all 7 days. We also assessed the risks and symptoms of participants with a risk for depression (based on CES-D>16) and suggested primary care office visits or made referrals per study protocol.

### Data Management and Analysis

EMA data were exported from the mEMA server to a comma-separated values file format. We entered the EMA and accelerometer data as well as the baseline surveys and anthropometric and clinical data into a database uploaded into SAS for analysis. We reviewed the data and corrected errors, missing data, outliers, and skewness and calculated the scale scores for the EMA responses. Descriptive analysis was used for demographic characteristics, anthropometric and clinical data, and the average values for EMA and PA data. Pearson and Spearman correlation coefficients were calculated at the individual level using the following variables: age, sex, BMI, CES-D, RES sum, MLD sum, annual income, education, blood pressure, body fat, racial identity, subjective social status, smoking and alcohol consumption, EMA survey data, and accelerometer data. EMA and PA data were scored for daily and individual (average in subject) levels. Intraclass correlation coefficients were calculated to quantify the proportions of total variance of PA explained by within- and between-person variances. Multilevel models for predicting PA (percentage sedentary time and percentage MVPA) were developed to examine the associations with EMA survey data (racial discrimination and microaggression) at the 2-hour interval (within-person), daily (within-person), and individual (between-person) levels. The models included within- and between-person levels of racial discrimination (model 1) or microaggression (model 2) with covariates (eg, age, sex, and BMI). Compound symmetry was used as a within-person correlation structure. Standardized coefficients were obtained using standardized outcomes and covariates with 0 mean and 1 SD.

## Results

### Overview

The mean response rate for EMA surveys was 83% (29/35; SD 16%), and the mean number of EMA responses per day was 4.0 (SD 1.2) out of a possible maximum of 5 per day. A total of 83.3% (10/12) of participants met the inclusion requirements for valid accelerometer data (≥10 hours/day wear time) and wore the accelerometer on the hip 6 out of 7 days. The mean EMA-reported daily racial discrimination was 0.61 (SD 0.85) per day, with a range of 0-2.28 (possible range: 0 to 10 times/day). Three participants reported no daily racial discrimination over the 7-day period (ie, their 7-day mean racial discrimination was 0). For the EMA-reported daily microaggression, the mean score was 50.26 (SD 18.11), with a range of 19.14-76.71 (possible range: 15-105/day).

Participant characteristics and descriptive statistics from the survey and anthropometric and clinical and accelerometer data are presented in Table 1. The mean age was 43.4 (SD 7.73) years. The majority worked full-time. Approximately 67% (8/12) had an annual income of less than US $60,000. The mean CES-D score was 21.08 (SD 8.36). The mean Black racial identity (centrality) was 5.21 (SD 1.46), indicating that most of our participants self-defined Black race as a core part of their self-concept. The mean subjective social status was 7.08, indicating that most rated their social status as high in the community. The mean BMI was 34.19 (SD 11.41) kg/cm^2^; approximately 42% (5/12) of the participants were obese. The mean MVPA was 18.5 minutes/day, and the mean sedentary time was 8.6 hours/day. Paired data, including both EMA and valid accelerometer data, resulted in a sample size of 9.

**Table 1:** Characteristics of the participants (n=12).

Characteristic		Value
Age (years), mean (SD)		43.4 (7.7)
Woman, n (%)		8 (67)
**Work, n (%)**		
	Working full-time	10 (83)
	Working part-time	2 (17)
**Annual income (US $), n (%)**		
	0-39,999	2 (17)
	40,000-59,999	6 (50)
	60,000-79,999	1 (8)
	80,000-99,999	2 (17)
	>100,000	1 (8)
**Education, n (%)**		
	Some high school	1 (8)
	Vocational or technical school	1 (8)
	Some college	5 (42)
	College graduate	5 (42)
**BMI (kg/m^2^)**		
	Mean (SD)	34.1 (11.4)
	18.5-24.9 (normal), n (%)	3 (25)
	25-29.9 (overweight), n (%)	4 (33)
	30-34.9 (class 1 obesity), n (%)	0 (0)
	35-39.9 (class 2 obesity), n (%)	1 (9)
	>40 (class 3 obesity), n (%)	4 (33)
Systolic blood pressure (mm Hg), mean (SD)		123.0 (16.1)
Diastolic blood pressure (mm Hg), mean (SD)		82.5 (13.0)
Total body fat (%), mean (SD)		38.8 (14.2)
Visceral fat (%), mean (SD)		10.8 (5.3)
RMAS^a^ (1-7), mean (SD)		4.5 (1.0)
EOD^b^ (0-9), mean (SD)		5.1 (2.5)
Racial identity (1-7), mean (SD)		5.2 (1.5)
**Depression>(by CES-D^c^; 0-60)**		
	Mean (SD)	21.1 (8.4)
	≥Cutoff value of 16, n (%)	8 (67)
	≥Cutoff value of 20, n (%)	5 (42)
Subjective social status (1-10), mean (SD)		7.1 (2.4)
**Smoking, n (%)**		
	No	11 (92)
	Yes	1 (8)
**Alcohol consumption, n (%)**		
	Never	3 (25)
	Monthly or less	5 (42)
	2-4 times a month	4 (33)
MVPA^d^ (min/day)^e^, mean (SD)		18.5 (16.3)
Total energy expenditure (kcal/day)^e^, mean (SD)		547.3 (280.2)
Sedentary time (hour/day)^e^, mean (SD)		8.6 (2.1)

^a^RMAS: Racial Microaggression Scale.

^b^EOD: Experiences of Discrimination.

^c^CES-D: Center for Epidemiological Studies Depression Scale.

^d^MVPA: moderate-to-vigorous physical activity.

^e^Indicates accelerometer data (n=9).

### Between-Persons Survey and EMA Analyses

In the bivariate analysis, using baseline surveys and anthropometric and clinical data, depressive symptoms were associated with major lifetime discrimination (*r*=0.58; *P*=.04) and a higher frequency of major lifetime discrimination (*r*=0.67; *P*=.04). Visceral fat was associated with diastolic blood pressure (*r*=0.62; *P*=.04) and sedentary time (*r*=0.73; *P*=.04) but was not associated with major lifetime discrimination. Income level was not significantly associated with Black racial identity (centrality; *r*=−0.26; *P*=.41).

Table 2 shows the bivariate correlations between the baseline sample characteristics and the average of the EMA-reported daily racial discrimination variables or PA variables. Greater EMA-reported daily racial discrimination was significantly associated with younger age (*r*=−0.75; *P*=.02). Black racial identity was not significantly associated with EMA-reported daily racial discrimination (*r*=0.21; *P*=.58) or microaggression (*r*=0.06; *P*=.88). Daily EMA-reported microaggression was associated with depressive symptoms (*r*=0.66; *P*=.05), past race-related events (*r*=0.82; *P*=.004), and major lifetime discrimination (*r*=0.78; *P*=.01). A higher total energy expenditure was significantly associated with less major lifetime discrimination (*r*=−0.92; *P*=.004). Less sedentary time was significantly associated with a stronger Black racial identity (*r*=−0.68; *P*=.04). More MVPA was significantly associated with lower levels of subjective social status (*r*=−0.75; *P*=.02).

**Table 2:** Correlations between demographic characteristics and the average scores of the ecological momentary assessment survey or physical activity.

Variables	EMA^a^ survey						Physical activity							
	Racial discrimination		Microaggression			Total energy expenditure				Percentage sedentary time			Percentage MVPA^b^	
	Correlation, *r*	*P* value	Correlation, *r*	*P* value	Correlation, *r*			*P* value	Correlation, *r*		*P* value	Correlation, *r*		*P* value
Age^c^	−*0.75*^d^	*.02*	−0.04	.92	0.42			.26	−0.01		.97	0.05		.89
Sex^c^	−0.39	.30	0.21	.59	−0.07			.86	0.24		.54	−0.31		.42
BMI^c^	0.09	.82	0.05	.90	0.65			.06	0.52		.15	0.23		.55
Depression (CES-D^e^)^c^	0.60	.08	*0.66*	*.05*	−0.53			.14	0.46		.22	−0.53		.14
Annual income^f^	−0.45	.22	−0.04	.91	−0.09			.82	0.47		.20	−0.31		.42
Education^f^	−0.30	.42	−0.33	.38	0.52			.15	0.19		.63	0.33		.38
Systolic blood pressure^c^	0.32	.41	0.04	.92	0.30			.43	0.64		.06	−0.45		.23
Diastolic blood pressure^c^	0.43	.24	0.00	.99	0.30			.44	0.61		.08	−0.23		.55
Total fat^c^	−0.22	.56	0.10	.79	0.52			.15	0.5		.17	0.00		.99
Racial identify^f^	0.21	.58	0.06	.88	−0.02			.95	−*0.68*		*.04*	0.04		.91
Subjective social status^f^	−0.14	.72	0.30	.44	−0.22			.57	0.22		.57	−*0.75*		*.02*
Smoking^f^	0.42	.26	−0.14	.72	−0.14			.72	0.00		.99	0.14		.72
Alcohol consumption^f^	−0.13	.74	−0.10	.82	−0.59			.09	−0.54		.13	0.00		.99
Past RES^g^ (RES sum)^c^	0.52	.15	*0.82*	*.004*	−0.60			.09	−0.18		.64	−0.12		.75
MLD^h^ scale (MLD sum)^c^	0.40	.28	*0.78*	*.01*	−*0.92*			*.004*	−0.11		.77	−0.59		.09

^a^EMA: ecological momentary assessment.

^b^MVPA: moderate-to-vigorous physical activity.

^c^Indicates Pearson correlation coefficients.

^d^Italicized values denote significance.

^e^CES-D: Center for Epidemiological Studies Depression Scale.

^f^Indicates Spearman correlation coefficient.

^g^RES: Race-Related Events Scale.

^h^MLD: Major Life Discrimination.

### Within- and Between-Person EMA Analyses

Intraclass correlation coefficients were calculated to represent the proportion of the total variance of the PA outcomes explained by the between-person levels. They were 0.54, 0.26, and 0.66 for total energy expenditure, sedentary time, and MVPA, respectively.

The within-person interval-level analysis found that during the 2-hour windows in which people reported more perceived racial discrimination, they had moderately greater sedentary time (β=.30, SE 0.21; *P*=.18) and slightly more MVPA (β=.04, SE 0.13; *P*=.77). Similarly, during the 2-hour windows in which they reported more perceived microaggression, they had less sedentary time (β=−.11, SE 0.16; *P*=.51) and less MVPA (β=−.34, SE 0.18; *P*=.10). However, none of these relationships during the 2-hour windows reached statistical significance.

The within-person daily levels and between-person analyses are presented in Table 3. In the within-person daily-level analyses, the association of racial discrimination and sedentary time was significant (β=.30, SE 0.14; *P*=.03), indicating that during days when participants reported more perceived racial discrimination, they had moderately more sedentary time.

**Table 3:** Multilevel model of mean daily physical activity outcomes (multilevels: level 1=daily and level 2=subject).^a^

Predictor variables and level			Outcome variables											
			Total expenditure (kcals) daily				Percentage sedentary time daily					Percentage MVPA^b^ daily (log-transformed)		
			Coefficient (SE)		*P* value		Coefficient (SE)		*P* value		Coefficient (SE)			*P* value
**Model 1: racial discrimination**														
	Within-person	0.12 (0.12)		.31		*0.30 (0.14)* ^c^		*.03*		0.09 (0.11)			.41	
	Between-person	−0.30 (0.28)		.29		−0.07 (0.24)		.76		−0.07 (0.31)			.81	
**Model 2: microaggression**														
	Within-person	−0.05 (0.28)		.84		−0.05 (0.34)		.87		−0.25 (0.26)			.33	
	Between-person	−0.34 (0.36)		.34		0.13 (0.39)		.73		0.03 (0.39)			.93	

^a^The predictor and outcome variables were standardized for 0 mean (SD 1).

^b^MVPA: moderate-to-vigorous physical activity.

^c^Italicized values denote significance.

## Discussion

### Principal Findings

Perceived racial discrimination is a significant psychological stressor that is hypothesized to have negative mental and physical health consequences with potential interactions with unhealthy behaviors. The relationship between overall psychological stress level and PA using EMA and objective measures has been evaluated in the general population; however, in what we believe to be the first published study of its kind, we examined momentary- and daily-level perceived racial discrimination and PA levels using EMA and accelerometers in African Americans. We collected repeated real-time racial discrimination exposure data in the natural environment while simultaneously collecting objective measures of sedentary behaviors and PA among African Americans. We also demonstrated the utility and feasibility of EMA coupled with accelerometers in studying the relationship between daily racial discrimination and PA in African Americans. Conventional accelerometer protocols require only 4 valid days for a 7-day wear period to be considered valid [39,43]. Approximately 83% (10/12) of our participants met the inclusion requirement for valid accelerometer data (≥10 hours/day wear time) and wore the accelerometer 6 out of 7 days, and they also showed high adherence to the EMA protocol.

In the examination of within-person level data, on days when participants reported more perceived racial discrimination than usual (ie, higher than their personal mean), more sedentary time was observed in the accelerometer data. The between-person analysis did not duplicate this finding in our study. However, this is consistent with the findings of between-person analysis in a prior study examining the relationship between general psychological stress and sedentary behaviors in other populations: end-of-day general stress ratings were not associated with sedentary time in the between-person analysis (at the group level) [16]. The influence of stress on sedentary behavior varies according to the source of stress within individuals [16,44]. Heterogeneity in the effect of stress on the amount and pattern of sedentary behaviors has been documented; for example, argument-related stress was associated with increased sedentary time, whereas work-related stress was associated with decreased sedentary time [16,45]. Similarly, in a study of sexual and gender minority individuals, between-person associations of discriminatory experiences and substance use were not significant, whereas more discriminatory experiences were significantly associated with more nicotine, alcohol, and drug use within the person [21]. This highlights the potential limitations of between-person methods (nomothetic) that predominate in research and suggests that the within-person level (idiographic) precision health approach may be highly relevant to target reductions in sedentary time and other unhealthy behaviors [16,44].

An important advantage of the EMA methodology is its ability to examine the frequency of racial discrimination experiences in real time and assess the impact of the experiences in a microtemporal relationship (eg, repeated assessments across minutes or hours). In our study, participants reported, on average, 0.61 overt racial discrimination experiences per day, and most participants experienced substantial daily microaggressions. The reported frequency of racial discrimination varies widely across studies [46-49]. In earlier cross-sectional studies using retrospective measures, discrimination was reported to occur only infrequently [50]; however, recent studies using EMA or other types of daily diaries have revealed that discrimination may occur multiple times per day. For example, in a study using EMA, African American participants reported about 2 experiences per day of racism [20]. In another study using EMA among African American adolescents [22], participants reported 5 experiences of racial discrimination per day when comprehensive measures of racial discrimination were used, including social media, vicarious, and teasing experiences, along with the more commonly measured individual and general forms of racial discrimination.

In several studies of psychological stress, not specific to racial discrimination-related stress in the general population, episodic stress predicts less PA, more sedentary behaviors, and reduced total energy expenditure [15,51]. Consistent with these studies, we found that major lifetime discrimination (from a retrospective measure) was significantly associated with lower total energy expenditure measured by the accelerometer. However, EMA-reported microaggressions were not associated with PA outcomes in our within-or between-person analyses. The nonsignificant relationship may be because of the small sample size and lack of variability in terms of the frequency of microaggression experiences within and across days. Overall, our participants reported frequent daily microaggressions, which may not have had a significant impact on their daily PA levels. However, the observed effect size based on standardized β coefficients [52] suggests the need for more studies to examine the determinants of PA and sedentary behaviors with a larger sample size and a longer assessment period.

Consistent with other studies [46,53,54], retrospectively measured exposure to race-based discrimination over a lifetime (assessed at baseline) was significantly associated with more depressive symptoms and with more daily microaggression experiences measured by EMA. Given the different data collection methods (retrospective surveys vs EMA) in this study, we could not determine the temporal relationship between racial discrimination or microaggression and depressive symptoms, and the findings may reflect a reverse causal relationship (eg, people with more depressive symptoms or such traits may perceive more microaggression). However, lagged effects of racial discrimination on depressive symptoms in subsequent days were reported among African Americans and Hispanics or Latinos in other studies [49,55], suggesting that individuals may not easily or fully recover from discrimination, and racial discrimination may have lasting effects on mental health [50,56]. Taken together, our findings highlight the important association between racial discrimination and mental health. Furthermore, future studies examining additional psychological factors, such as traits and personality, are needed to determine both the concurrent and lagged effects of racial discrimination on health and health behaviors. Such studies may inform the development of individualized interventions that can buffer the harmful effects of racial discrimination on health.

### Strengths and Limitations

This study had several limitations. Although we found similar trends in within- or between-person effects on sedentary behaviors and PA, compared with other studies of general psychological stress, our small sample size offers limited evidence supporting racial discrimination as an antecedent to sedentary behaviors or PA. EMA minimizes recall bias and errors. However, it is also possible that our study findings may have been influenced by vigilance to discrimination from the repetitive assessment involved in EMA. In addition, the high CES-D scores observed in our participants may have influenced the associations with perceived racial discrimination or PA. Although findings are mixed, previous studies have shown that neighborhood environments such as walkability, safety, or crime were associated with individuals’ PA levels in the general population [57,58]. We obtained walkability (Walk Score) and crime index data based on participants’ zip codes (data not shown); however, the predominantly Black neighborhoods in our sample showed a lack of variability. Future studies with measures of social environment, segregation, and perceived neighborhood environments, in addition to objective built environments, would be helpful in understanding the relationship between PA and relevant correlates. Owing to the exploratory nature of our pilot study with the scarcity of EMA studies of racial discrimination, we conducted a 2-hour within-person, prompt-level analysis; however, assessment may need longer time frames to determine the association between racial discrimination and PA levels. In addition, using event-contingent sampling (ie, EMA is reported when a discrimination event occurs) may be helpful in determining the frequency of racial discrimination; one caveat is that it may not accurately measure events if many participants forget to report them (missing EMA). In addition, our study included only in-person and individual racial discrimination experiences. Including web-based (eg, communication in social media) and vicarious discrimination experiences (eg, watching traumatic videos of police brutality) may provide more valid frequency estimates [22,59]. Future efforts should include studies with a large sample, more extensive racial discrimination measures, and EMA sampling to determine the optimal frequency of EMA to accurately capture discriminatory experiences and to examine its relationship with health behaviors.

Despite these limitations, this study provides valuable insights into examining the within-person effects of racial discrimination on health behaviors and suggests the need to examine a more complex relationship between racial discrimination and lifestyle behaviors with time-varying factors.

There is a growing emphasis on within-person examination of health behaviors and psychosocial correlates and on the importance of leveraging these data to develop personalized, *just-in-time* interventions [50,60]. Examining this daily process using a within-person approach has the potential to elucidate the mechanisms of which racial discrimination may have on health and health behaviors and to guide the development of personalized interventions for increasing PA and decreasing depressive symptoms in racial ethnic minorities.

### Conclusions

In conclusion, the results of this study highlight the utility and feasibility of a within-person approach to target reductions in sedentary time and improvements in PA associated with daily racial discrimination by using EMA and an objective measure of PA. Further studies are needed to confirm the observed findings in light of the limitations of this study, including its small sample size. A precision health approach that incorporates between-person associations and accounts for within-person variations in the relationship between racial discrimination and health behaviors is warranted to mitigate race-based health disparities.

## Abbreviations

CES-D: Center for Epidemiological Studies Depression Scale
EMA: ecological momentary assessment
EOD: Experiences of Discrimination
MLD: Major Life Discrimination
MVPA: moderate-to-vigorous physical activity
PA: physical activity
RES: Race-Related Events Scale
RMAS: Racial Microaggression Scale
VM: vector magnitude
